# Say what you please? Really?

**DOI:** 10.3389/fpsyg.2013.00237

**Published:** 2013-04-26

**Authors:** Lyn Frazier

**Affiliations:** Department of Linguistics, University of Massachusetts AmherstAmherst, MA, USA

Gennari and MacDonald (2009) presented evidence that reading times in comprehension are influenced by the likelihood of a speaker choosing a particular structure in production. It's an important and interesting paper. From this they propose the PDC (Production Distribution Comprehension): choices made to ease planning in production give rise to distributional patterns in the input, which in turn influence comprehension. The present paper suggests maybe everything (language production, acquisition, parsing, and language typology) works this way and seems to imply that therefore we might not need abstract syntactic representations, recursion, a language acquisition device, etc., at least not to explain processing difficulty. Maybe, but we might want more evidence. In addition to the subject- vs. object-relative clause distinction that motivated the PDC, a second case is discussed in the current paper: modifiers that may attach low or high. But the argumentation only shows that a production account is in principle possible, it doesn't say why it's any better than available comprehension accounts.

PDC is usually discussed as a “view” or “perspective.” But when the author suggests it's mechanistic, it sounds more like an empirical claim. If it is an empirical claim, then one might want to know how distributional patterns are discovered in the input, how general they are, how they are stored, and how they are utilized. After all, on the PDC model, that's where all the action is, at least for the comprehension system. It might also be germane to figure out whether the statistics are gathered separately for different speakers, different dialects, different registers. Also, it might be good to provide evidence that it IS the statistics themselves that guide expectations in comprehension, and not implicit knowledge of how the production system works.

As for the PDC perspective, let's endorse it. Let's look for production-based explanations, and any other explanations we can construct, if they are precise enough to make predictions.

Does the PDC make predictions? It might if speakers can say what they please. But in fact the grammar of the language will dictate what constitutes a phrase, what order is permitted or required, whether ellipsis is possible, and so forth. And the grammar demonstrably is NOT just the summation of past favored production choices but, in addition to whatever constraints acquisition or comprehension may impose, convention and historical accident also come into play, and these various factors interact in complex ways. So, at best, PDC applies when the grammar offers more than one option for the same message. To take a well-known example, in sentences containing a dative verb, Double Object structures (DO) and Prepositional Object structures (PO) are largely synonymous. Maybe in such cases principles like Easy First apply. Although Easy First is not well-defined, clearly on any tenable version of it, phrases already “given” in discourse will count as “easy.” So the prediction is that given should appear before new, listeners should store these statistics, and comprehenders should find these structures easier when given appears before new. So there is a prediction. The problem in this case, however, concerns the facts. For DO, the prediction appears to be correct. But for PO, it is not (Clifton and Frazier, 2004; Brown et al., 2012). What should we conclude from this? That PDC is confirmed (by DO structures)? That PDC is disconfirmed (by PO structures)? That in precisely the case of interest, where the grammar permits more than one option, whatever is going on is more complex than PDC countenances?

Let's take another example. Perhaps the dative alternation is misleading for some reason.

Take extraposition from subject (1b) and from object (2b). Apparently we could explain the ease of (1b) with “Easy First”—the most important of the three core production planning principles since “Plan reuse/priming” will depend on context, and Reduce interference will depend on lexical choices. But “Easy First” would seem to predict no advantage for extraposition from object (2b) since the object is last regardless of whether the object is extraposed, as in (2b), or not extraposed (2a). Nevertheless, intuitively, and in pilot data of Emily Westland (2012), (2b) is markedly better than (2a). Like the dative alternation example, this suggests a PDC approach may be a bit too simple.

1.
  - That Max left early bothered me.
  - It bothered me that Max left early. (Extraposition from subject)
2.
  - I hated that Max left early.
  - I hated it that Max left early. (Extraposition from object)
  Let's consider another example. Take the repeatedly demonstrated finding that speakers will include optional constituents (e.g., the complementizer *that*) at points of high complexity in production (Rohdenburg, 1996; Jaeger, 2010). What does the PDC predict? Should listeners and readers have difficulty with (3a) because the upcoming constituent is simple?
3.
  - I know that Sam tripped.
  - I know Sam tripped

In other words, the input in (3a) is not of the form the speaker would have been most likely to choose so perhaps the PDC predicts comprehension difficulty.

Turning to the second major production claim “Plan reuse/frequency/priming,” it is important to recall that empirical evidence supports the existence of abstract syntactic representations (Bock, 1989; Pickering and Ferreria, 2008; Ivanova et al., 2012, for example). If abstract representations did not exist, priming would yield sequences like *Hear what I say? Hear what who say?* (agreement error intended), or worse *Hear what I say, hear what I say, hear what I say*….

As for the third production claim, Reduce interference, there is explicit memory research showing the existence of similarity interference in memory. The majority of research in this area seems to show interference from *grammatical* features. Further, in production, in cases involving number where grammatical and conceptual features have both been manipulated, it is primarily the grammatical features that mattered (Bock and Eberhard, 1993). This raises the possibility that to a large extent it is the grammar that provides the similarity metric.

As for PDC predictions about typology, without discussion of grammar, any typological generalizations would seem by definition to be limited to superficial generalizations. And what about language as a system? Is there room in the PDC view for an item to tend to behave like other members of its class, thereby shaping a language? For example, for a noun to tend to share the distribution of other nouns, to pluralize if it is a count noun, to be input to morphological rules that take nouns as input? Or for a phrase used as a topic in a particular sentence, to share the distributional properties of other topics, and their prosodic and interpretive properties? Or would that be a “powerful force” not attributable to production and therefore evidence against the PDC?

To sum up, in terms of the characterization of production, the proposed principles, operations, and representations are not sufficiently well-defined (what counts as “easy?” what counts as “first”—earliness in planning or precedence in linear order?). In terms of distribution, it is not clear what is counted, why, nor are the implied mechanisms spelled out. As for the claim that comprehension ease is determined by production ease (mediated by distribution), only one case study has been presented that shows a production-based explanation is best, rather than merely possible. Against this there are several apparently incorrect predictions of PDC about comprehension difficulty in datives, argument extraposition, and complementizer use.

Is there a productive way forward that goes beyond simply tallying up the columns of the scoreboard? In my view, yes. MacDonald's thoughtful paper could be the first step toward an explanatory theory of sub-categorization of lexical items, i.e., sub-classification of items based on the type of argument and the actual arguments with which a lexical head of phrase co-occurs. All theories of comprehension rely on stored sub-categorization information without much concern for precisely what information is encoded, where it came from, or even the basic question of whether the lexical-syntactic interface in comprehension extends beyond what's captured by sub-categorization.
